# The genome sequence of the Mouse Moth, *Amphipyra tragopoginis* (Clerck 1759)

**DOI:** 10.12688/wellcomeopenres.18946.1

**Published:** 2023-02-03

**Authors:** Douglas Boyes, University of Oxford and Wytham Woods Genome Acquisition Lab, Darwin Tree of Life Barcoding collective, Wellcome Sanger Institute Tree of Life programme, Wellcome Sanger Institute Scientific Operations: DNA Pipelines collective, Tree of Life Core Informatics collective, Peter W.H. Holland, Darwin Tree of Life Consortium

**Affiliations:** 1 UK Centre for Ecology and Hydrology, Wallingford, Oxfordshire, UK; 2 University of Oxford, Oxford, Oxfordshire, UK

**Keywords:** Amphipyra tragopoginis, the Mouse Moth, genome sequence, chromosomal, Lepidoptera

## Abstract

We present a genome assembly from an individual male *Amphipyra tragopoginis* (the Mouse Moth; Arthropoda; Insecta; Lepidoptera; Noctuidae). The genome sequence is 806 megabases in span. Most of the assembly is scaffolded into 31 chromosomal pseudomolecules, including the assembled Z sex chromosome. The mitochondrial genome has also been assembled and is 15.3 kilobases in length. Gene annotation of this assembly on Ensembl has identified 13,359 protein coding genes.

## Species taxonomy

Eukaryota; Metazoa; Ecdysozoa; Arthropoda; Hexapoda; Insecta; Pterygota; Neoptera; Endopterygota; Lepidoptera; Glossata; Ditrysia; Noctuoidea; Noctuidae; Amphipyrinae; *Amphipyra*; *Amphipyra tragopoginis* (Clerck 1759) (NCBI:txid689058).

## Background

The Mouse moth, *Amphipyra tragopoginis* (Clerck 1759), is a moth in the family Noctuidae found across central and northern Europe, with scattered records from Asia ( GBIF Secretariat, 2021). The species has also been recorded in the United States and Canada, where it is has probably been accidentally introduced ( Forbes, 1954; Lotts & Naberhaus, 2021). The forewings of the adult are a uniform shiny grey marked with three small black dots forming an elongated triangle; this simple pattern is distinctive among moths in the UK. The adult moths have an unusual behavioural trait that suits the common name well: when disturbed the moth often scuttles away, instead of flying. Despite the aversion to flight as an escape response, *A tragopoginis* was abundant in a study of aerial insects sampled using suction traps at a height of 12 metres and is clearly a strong flyer ( Wood *et al.*, 2009). The moth seems to fly mostly in the early part of the night, just after darkness, and favours warm nights up to 21°C ( Taylor, 1963).

In the UK, the adult moth is on the wing from July to September, and although sometimes caught at light it is more reliably attracted to sugary baits. Since most moth recording uses light traps, it is therefore possible that the moth is under recorded. Even so, standardised trapping methods conducted annually across the UK suggest the moth is declining in abundance, with a decrease of over 80% from 1970 to 2016 ( Randle *et al.*, 2019). The decline is unlikely to be connected to food plant availability, since the larvae are polyphagous and feed on the leaves of a large range of trees, shrubs and herbaceous plants.

A genome sequence from *Amphipyra tragopoginis* will be useful for research into wing pattern evolution and behavioural adaptations, and more generally for comparative studies across the Lepidoptera.

## Genome sequence report

**Figure 1.**
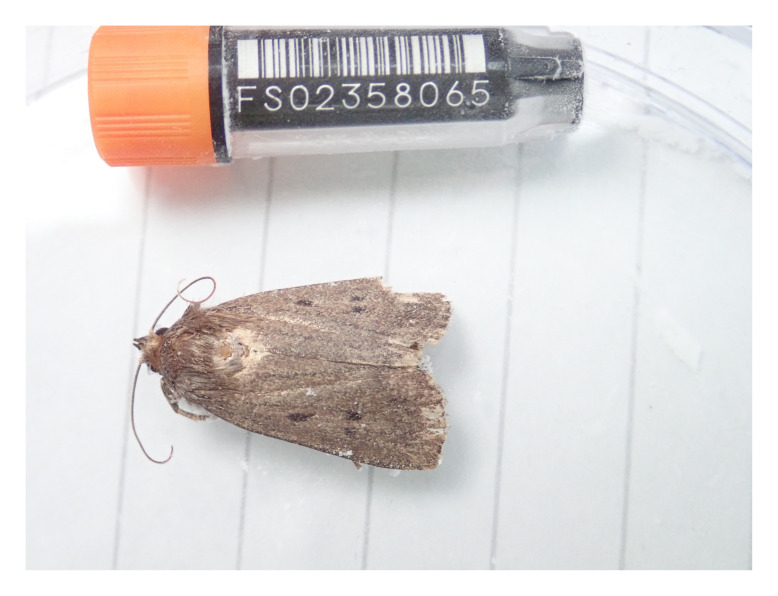
Photograph of the *Amphipyra tragopoginis* (ilAmpTrag2) specimen used for genome sequencing.

The genome was sequenced from one male *Amphipyra tragopoginis* ( Figure 1) collected from Wytham Woods, UK (latitude 51.77, longitude –1.34). A total of 41-fold coverage in Pacific Biosciences single-molecule HiFi long reads and 49-fold coverage in 10X Genomics read clouds were generated. Primary assembly contigs were scaffolded with chromosome conformation Hi-C data. Manual assembly curation corrected 23 missing or mis-joins and removed four haplotypic duplications, reducing the assembly length by 1.36% and the scaffold number by 23.81%, and increasing the scaffold N50 by 4.17%.

**Figure 2.**
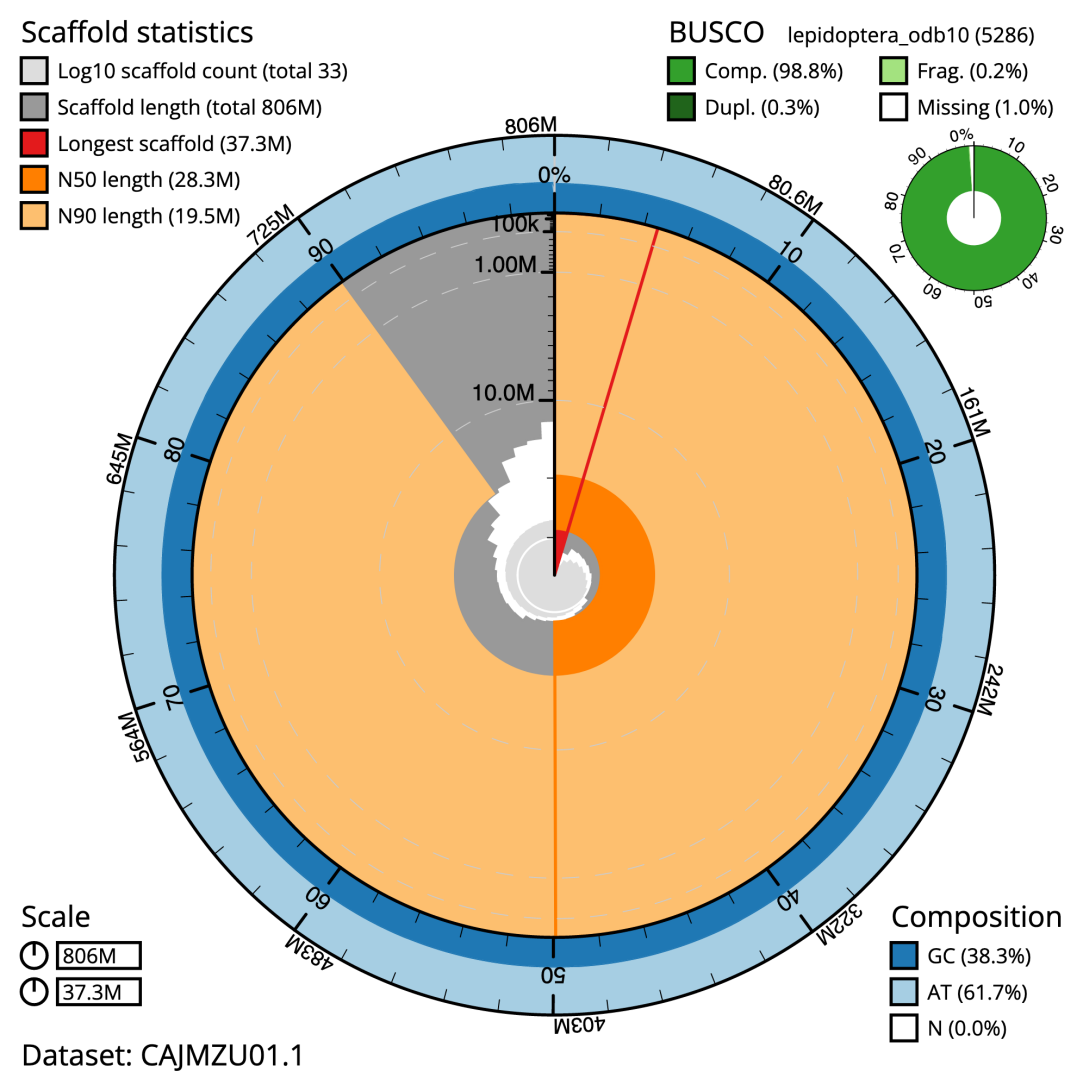
Genome assembly of *Amphipyra tragopoginis*, ilAmpTrag2.1: metrics. The BlobToolKit Snailplot shows N50 metrics and BUSCO gene completeness. The main plot is divided into 1,000 size-ordered bins around the circumference with each bin representing 0.1% of the 805,668,602 bp assembly. The distribution of scaffold lengths is shown in dark grey with the plot radius scaled to the longest scaffold present in the assembly (37,340,338 bp, shown in red). Orange and pale-orange arcs show the N50 and N90 scaffold lengths (28,301,216 and 19,491,276 bp), respectively. The pale grey spiral shows the cumulative scaffold count on a log scale with white scale lines showing successive orders of magnitude. The blue and pale-blue area around the outside of the plot shows the distribution of GC, AT and N percentages in the same bins as the inner plot. A summary of complete, fragmented, duplicated and missing BUSCO genes in the lepidoptera_odb10 set is shown in the top right. An interactive version of this figure is available at https://blobtoolkit.genomehubs.org/view/ilAmpTrag2.1/dataset/CAJMZU01.1/snail.

The final assembly has a total length of 805.7 Mb in 32 sequence scaffolds with a scaffold N50 of 28.3 Mb ( Table 1). Most (99.99%) of the assembly sequence was assigned to 31 chromosomal-level scaffolds, representing 30 autosomes and the Z sex chromosome. Chromosome-scale scaffolds confirmed by the Hi-C data are named in order of size ( Figure 2– Figure 5; Table 2). The assembly has a BUSCO v5.3.2 ( Manni *et al.*, 2021) completeness of 98.8% using the lepidoptera_odb10 reference set. While not fully phased, the assembly deposited is of one haplotype. Contigs corresponding to the second haplotype have also been deposited.

**Table 1.** Genome data for *Amphipyra tragopoginis*, ilAmpTrag2.1.

Project accession data		
Assembly identifier	ilAmpTrag2.1	
Species	*Amphipyra tragopoginis*	
Specimen	ilAmpTrag2	
NCBI taxonomy ID	689058	
BioProject	PRJEB42948	
BioSample ID	SAMEA7520175	
Isolate information	ilAmpTrag2; head/thorax (PacBio and 10X); abdomen (Hi-C) ilAmpTrag1; abdomen (RNA-Seq)	
Assembly metrics *		*Benchmark*
Consensus quality (QV)	58.1	*≥ 50*
*k*-mer completeness	100%	*≥ 95%*
BUSCO **	C:98.8%[S:98.5%,D:0.3%], F:0.2%,M:1.0%,n:5,286	*C ≥ 95%*
Percentage of assembly mapped to chromosomes	99.99%	*≥ 95%*
Sex chromosomes	ZZ	*localised homologous pairs*
Organelles	Mitochondrial genome assembled	*complete single alleles*
Raw data accessions		
PacificBiosciences SEQUEL II	ERR6544654, ERR7254633	
10X Genomics Illumina	ERR6054389–ERR6054392	
Hi-C Illumina	ERR6054393	
PolyA RNA-Seq Illumina	ERR6286711, ERR6286712	
Genome assembly		
Assembly accession	GCA_905220435.1	
*Accession of alternate haplotype*	GCA_905220425.1	
Span (Mb)	805.7	
Number of contigs	54	
Contig N50 length (Mb)	23.5	
Number of scaffolds	32	
Scaffold N50 length (Mb)	28.3	
Longest scaffold (Mb)	37.3	
Genome annotation		
Number of protein-coding genes	13,359	
Number of non-coding genes	2,680	
Number of gene transcripts	23,574	

* Assembly metric benchmarks are adapted from column VGP-2020 of “Table 1: Proposed standards and metrics for defining genome assembly quality” from ( Rhie *et al.*, 2021). ** BUSCO scores based on the lepidoptera_odb10 BUSCO set using v5.3.2. C = complete [S = single copy, D = duplicated], F = fragmented, M = missing, n = number of orthologues in comparison. A full set of BUSCO scores is available at https://blobtoolkit.genomehubs.org/view/ilAmpTrag2.1/dataset/CAJMZU01.1/busco.

**Figure 3.**
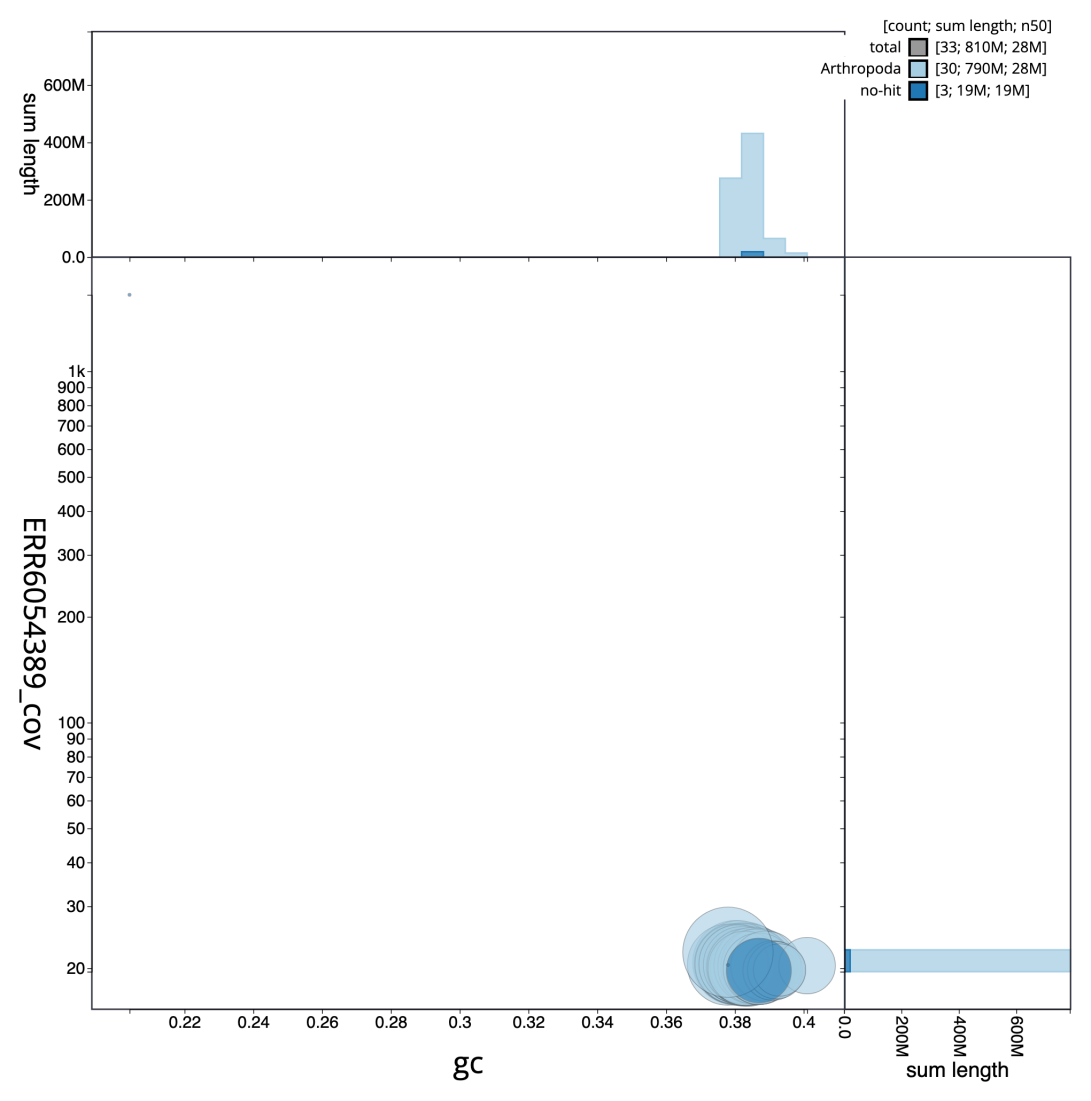
Genome assembly of *Amphipyra tragopoginis*, ilAmpTrag2.1: GC coverage. BlobToolKit GC-coverage plot. Scaffolds are coloured by phylum. Circles are sized in proportion to scaffold length. Histograms show the distribution of scaffold length sum along each axis. An interactive version of this figure is available at https://blobtoolkit.genomehubs.org/view/ilAmpTrag2.1/dataset/CAJMZU01.1/blob.

**Figure 4.**
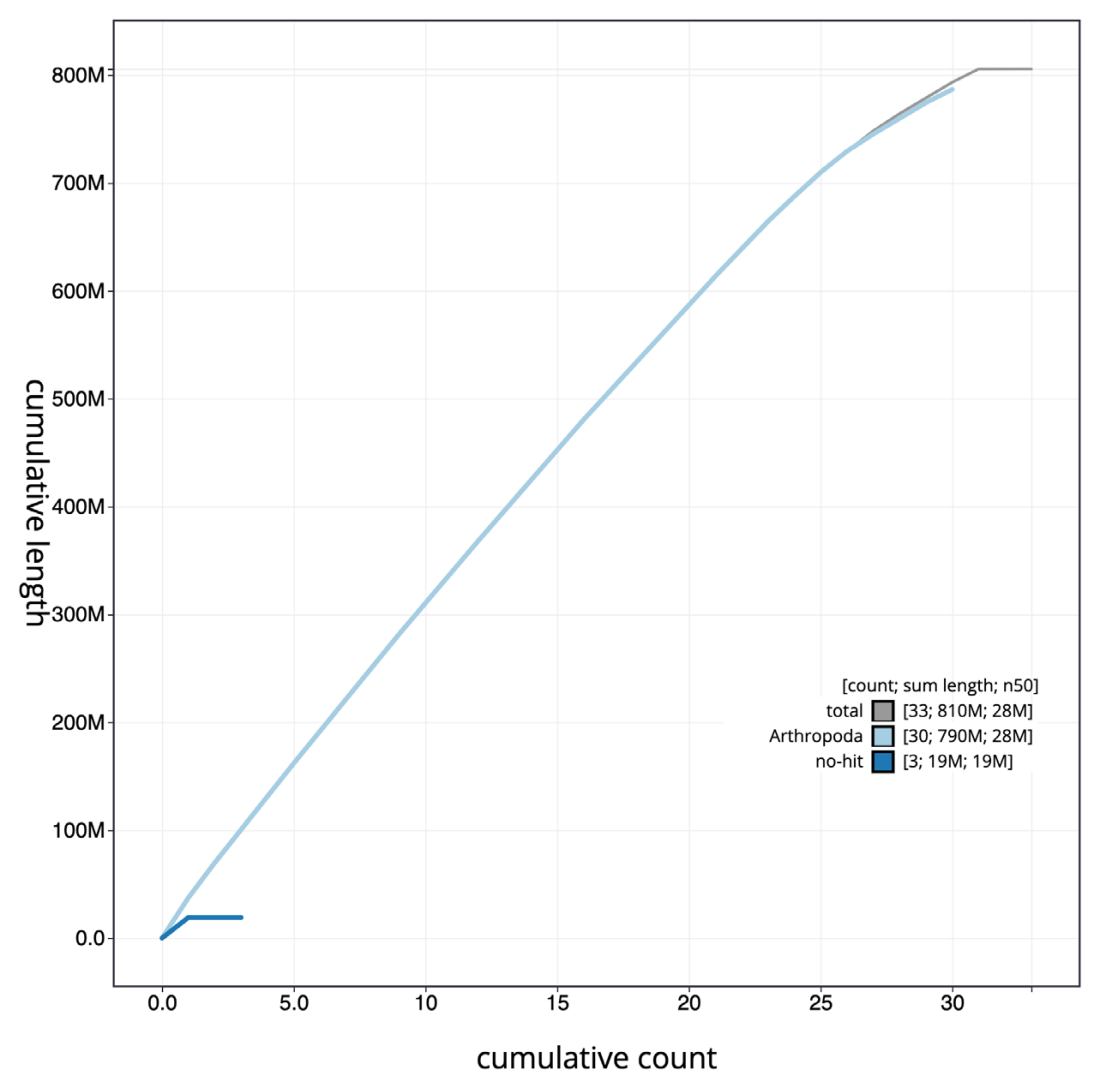
Genome assembly of *Amphipyra tragopoginis*, ilAmpTrag2.1: cumulative sequence. BlobToolKit cumulative sequence plot. The grey line shows cumulative length for all scaffolds. Coloured lines show cumulative lengths of scaffolds assigned to each phylum using the buscogenes taxrule. An interactive version of this figure is available at https://blobtoolkit.genomehubs.org/view/ilAmpTrag2.1/dataset/CAJMZU01.1/cumulative.

**Figure 5.**
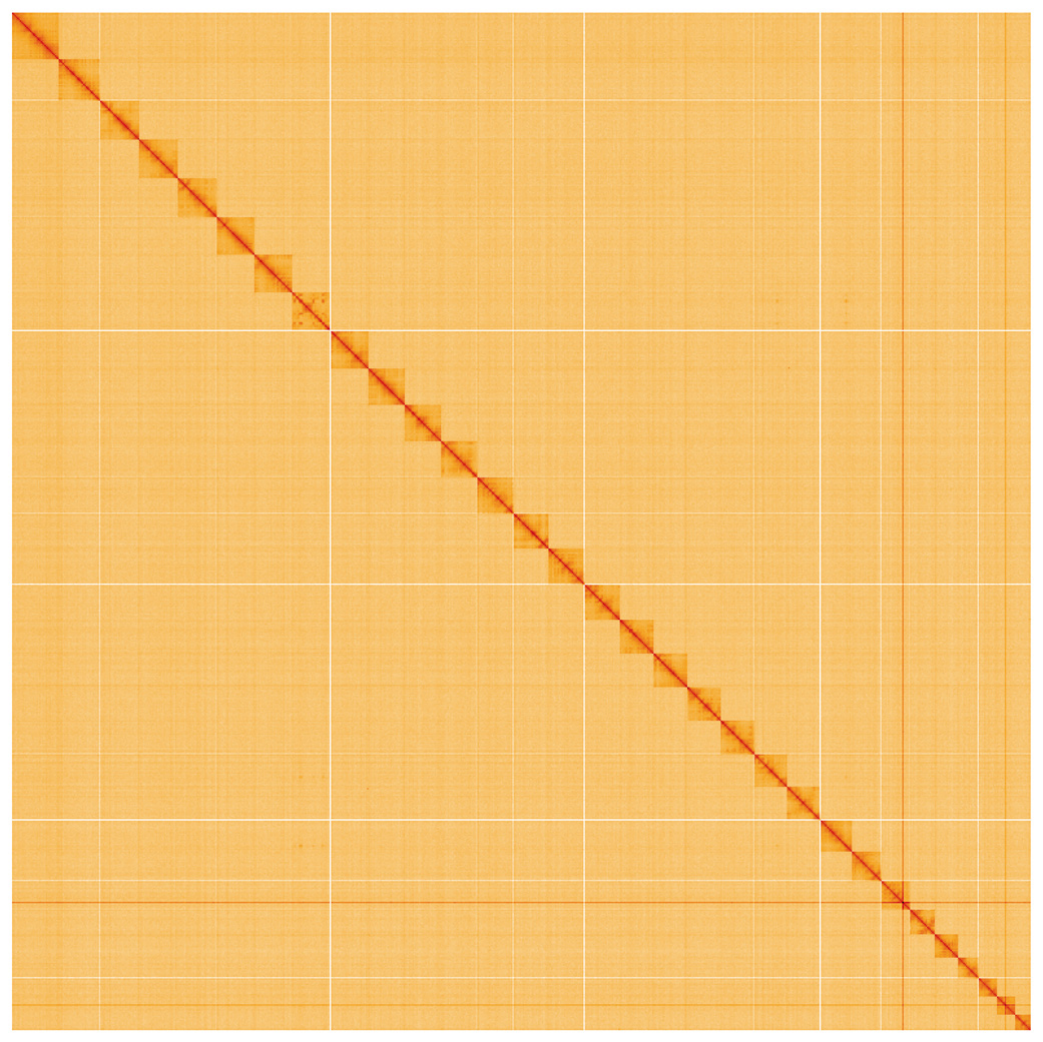
Genome assembly of *Amphipyra tragopoginis*, ilAmpTrag2.1: Hi-C contact map. Hi-C contact map of the ilAmpTrag2.1 assembly, visualised using HiGlass. Chromosomes are shown in order of size from left to right and top to bottom. An interactive version of this figure may be viewed at https://genome-note-higlass.tol.sanger.ac.uk/l/?d=H5VwgKcTTXuK2oXclYTjwA.

**Table 2.** Chromosomal pseudomolecules in the genome assembly of *Amphipyra tragopoginis*, ilAmpTrag2.

INSDC accession	Chromosome	Size (Mb)	GC%
HG991992.1	1	32.39	38
HG991993.1	2	30.96	38.3
HG991994.1	3	30.73	38.3
HG991995.1	4	30.52	38.1
HG991996.1	5	30.24	38.1
HG991997.1	6	29.91	37.8
HG991998.1	7	29.83	38.4
HG991999.1	8	29.81	38
HG992000.1	9	29.07	38.2
HG992001.1	10	28.79	38
HG992002.1	11	28.46	38.3
HG992003.1	12	28.3	38.4
HG992004.1	13	28.3	38.1
HG992005.1	14	28.11	38.1
HG992006.1	15	27.7	38.3
HG992007.1	16	26.86	38.4
HG992008.1	17	26.78	38.2
HG992009.1	18	26.55	38.3
HG992010.1	19	26.42	38.5
HG992011.1	20	26.23	38.7
HG992012.1	21	25.84	38.6
HG992013.1	22	25.11	38.3
HG992014.1	23	23.2	38.7
HG992015.1	24	22.36	38.9
HG992016.1	25	19.49	38.7
HG992017.1	26	18.96	38.7
HG992018.1	27	15.95	39.1
HG992019.1	28	14.79	39.2
HG992020.1	29	14.36	40.1
HG992021.1	30	12.29	39.3
HG991991.1	Z	37.34	37.8
HG992022.1	MT	0.02	20.2
-	unplaced	0.02	37.8

## Genome annotation report

The *A. tragopoginis* GCA_905220435.1 assembly was generated using the Ensembl rapid annotation pipeline ( Table 1; https://rapid.ensembl.org/Amphipyra_tragopoginis_GCA_905220435.1/). The resulting annotation includes 13359 protein coding genes with an average length of 20,732.64 bp and an average coding length of 1,473.70 bp, and 2680 non-protein coding genes. There is an average of 7.05 exons and 6.05 introns per canonical protein coding transcript, with an average intron length of 2,884.07. A total of 4,772 gene loci have more than one associated transcript.

## Methods

### Sample acquisition and nucleic acid extraction

Two *A. tragopoginis* specimens (ilAmpTrag1 and ilAmpTrag2) were collected in Wytham Woods, Oxfordshire (biological vice-county: Berkshire), UK (latitude 51.77, longitude –1.34) on 24 August 2019 using a light trap. The specimens were collected and identified by Douglas Boyes (University of Oxford) and snap-frozen on dry ice.

DNA was extracted at the Tree of Life laboratory, Wellcome Sanger Institute (WSI). The ilAmpTrag2 sample was weighed and dissected on dry ice with tissue set aside for Hi-C sequencing. Head and thorax tissue was cryogenically disrupted to a fine powder using a Covaris cryoPREP Automated Dry Pulveriser, receiving multiple impacts. High molecular weight (HMW) DNA was extracted using the Qiagen MagAttract HMW DNA extraction kit. Low molecular weight DNA was removed from a 20 ng aliquot of extracted DNA using 0.8X AMpure XP purification kit prior to 10X Chromium sequencing; a minimum of 50 ng DNA was submitted for 10X sequencing. HMW DNA was sheared into an average fragment size of 12–20 kb in a Megaruptor 3 system with speed setting 30. Sheared DNA was purified by solid-phase reversible immobilisation using AMPure PB beads with a 1.8X ratio of beads to sample to remove the shorter fragments and concentrate the DNA sample. The concentration of the sheared and purified DNA was assessed using a Nanodrop spectrophotometer and Qubit Fluorometer and Qubit dsDNA High Sensitivity Assay kit. Fragment size distribution was evaluated by running the sample on the FemtoPulse system.

RNA was extracted from abdomen tissue of ilAmpTrag1 in the Tree of Life Laboratory at the WSI using TRIzol, according to the manufacturer’s instructions. RNA was then eluted in 50 μl RNAse-free water and its concentration assessed using a Nanodrop spectrophotometer and Qubit Fluorometer using the Qubit RNA Broad-Range (BR) Assay kit. Analysis of the integrity of the RNA was done using Agilent RNA 6000 Pico Kit and Eukaryotic Total RNA assay.

### Sequencing

Pacific Biosciences HiFi circular consensus and 10X Genomics read cloud DNA sequencing libraries were constructed according to the manufacturers’ instructions. Poly(A) RNA-Seq libraries were constructed using the NEB Ultra II RNA Library Prep kit. DNA and RNA sequencing were performed by the Scientific Operations core at the WSI on Pacific Biosciences SEQUEL II (HiFi), Illumina HiSeq 4000 (RNA-Seq) and HiSeq X Ten (10X) instruments. Hi-C data were also generated from abdomen tissue of ilAmpTrag2 using the Arima v2 kit and sequenced on the HiSeq X Ten instrument.

### Genome assembly

Assembly was carried out with Hifiasm ( Cheng *et al.*, 2021) and haplotypic duplication was identified and removed with purge_dups ( Guan *et al.*, 2020). One round of polishing was performed by aligning 10X Genomics read data to the assembly with Long Ranger ALIGN, calling variants with freebayes ( Garrison & Marth, 2012). The assembly was then scaffolded with Hi-C data ( Rao *et al.*, 2014) using SALSA2 ( Ghurye *et al.*, 2019). The assembly was checked for contamination and corrected using the gEVAL system ( Chow *et al.*, 2016) as described previously ( Howe *et al.*, 2021). Manual curation was performed using gEVAL, HiGlass ( Kerpedjiev *et al.*, 2018) and Pretext ( Harry, 2022). The mitochondrial genome was assembled using MitoHiFi ( Uliano-Silva *et al.*, 2022), which performed annotation using MitoFinder ( Allio *et al.*, 2020). The genome was analysed and BUSCO scores generated within the BlobToolKit environment ( Challis *et al.*, 2020). Table 3 contains a list of all software tool versions used, where appropriate.

**Table 3.** Software tools and versions used.

Software tool	Version	Source
BlobToolKit	3.5.0	Challis *et al.*, 2020
freebayes	1.3.1-17-gaa2ace8	Garrison & Marth, 2012
gEVAL	N/A	Chow *et al.*, 2016
Hifiasm	0.12	Cheng *et al.*, 2021
HiGlass	1.11.6	Kerpedjiev *et al.*, 2018
Long Ranger ALIGN	2.2.2	https://support.10xgenomics.com/genome-exome/software/pipelines/latest/advanced/other-pipelines
MitoHiFi	1	Uliano-Silva *et al.*, 2022
PretextView	0.2	Harry, 2022
purge_dups	1.2.3	Guan *et al.*, 2020
SALSA	2.2	Ghurye *et al.*, 2019

### Genome annotation

The Ensembl gene annotation system ( Aken *et al.*, 2016) was used to generate annotation for the *A. tragopoginis* assembly GCA_905220435.1. Annotation was created primarily through alignment of transcriptomic data to the genome, with gap filling via protein to-genome alignments of a select set of proteins from UniProt ( UniProt Consortium, 2019).

### Ethics/compliance issues

The materials that have contributed to this genome note have been supplied by a Darwin Tree of Life Partner. The submission of materials by a Darwin Tree of Life Partner is subject to the Darwin Tree of Life Project Sampling Code of Practice. By agreeing with and signing up to the Sampling Code of Practice, the Darwin Tree of Life Partner agrees they will meet the legal and ethical requirements and standards set out within this document in respect of all samples acquired for, and supplied to, the Darwin Tree of Life Project. Each transfer of samples is further undertaken according to a Research Collaboration Agreement or Material Transfer Agreement entered into by the Darwin Tree of Life Partner, Genome Research Limited (operating as the Wellcome Sanger Institute), and in some circumstances other Darwin Tree of Life collaborators.

## Data Availability

European Nucleotide Archive: *Amphipyra tragopoginis* (mouse moth), genomic and transcriptomic data). Accession number PRJEB42948; https://identifiers.org/ena.embl/PRJEB42948. ( Wellcome Sanger Institute, 2021) The genome sequence is released openly for reuse. The *Amphipyra tragopoginis* genome sequencing initiative is part of the Darwin Tree of Life (DToL) project. All raw sequence data and the assembly have been deposited in INSDC databases. Raw data and assembly accession identifiers are reported in Table 1.

## Author information

Members of the University of Oxford and Wytham Woods Genome Acquisition Lab are listed here: https://doi.org/10.5281/zenodo.4789928.

Members of the Darwin Tree of Life Barcoding collective are listed here: https://doi.org/10.5281/zenodo.4893703.

Members of the Wellcome Sanger Institute Tree of Life programme are listed here: https://doi.org/10.5281/zenodo.4783585.

Members of Wellcome Sanger Institute Scientific Operations: DNA Pipelines collective are listed here: https://doi.org/10.5281/zenodo.4790455.

Members of the Tree of Life Core Informatics collective are listed here: https://doi.org/10.5281/zenodo.5013541.

Members of the Darwin Tree of Life Consortium are listed here: https://doi.org/10.5281/zenodo.4783558.
