# Supplementary material for: The genome sequence of the Mouse Moth, Amphipyra tragopoginis (Clerck 1759)
Source: Wellcome Open Res. 2023 Feb 3;8:54. [Version 1] doi: 10.12688/wellcomeopenres.18946.1 (PMC10285349; doi:10.12688/wellcomeopenres.18946.1)
Supplement: Peer review history

# Peer review: Reviewer response for version 1

**DOI:** 10.21956/wellcomeopenres.21006.r59185

**Published:** 2023-06-21

**Authors:** Hanamant Gadad

**Affiliations:** 1 Central Tasar Research and Training Institute, Central Silk Board, Ministry of Textiles, Ranchi, India

Findings of the research article "The genome sequence of the Mouse Moth, *Amphipyra tragopoginis* (Clerck 1759)", by Boyes *et al.; *provides comprehensive information on genome assembly and annotation on scientifically important lepidopteran species.

 Methods used are adequately described which allows the readers to understand the study and results are presented clearly and briefly and the study is well discussed by providing valuable insights into the implications of the findings. Overall the study significantly contributes to the field of Lepidoptera genomics and will serve as a valuable resource for future studies in various biological disciplines.

Are sufficient details of methods and materials provided to allow replication by others?

Yes

Is the rationale for creating the dataset(s) clearly described?

Yes

Are the datasets clearly presented in a useable and accessible format?

Yes

Are the protocols appropriate and is the work technically sound?

Yes

Reviewer Expertise:

Insecticide resistance, Insect chemical ecology, DNA barcoding, Lepidopteran ecology and conservation

I confirm that I have read this submission and believe that I have an appropriate level of expertise to confirm that it is of an acceptable scientific standard.

# Peer review: Reviewer response for version 1

**DOI:** 10.21956/wellcomeopenres.21006.r54705

**Published:** 2023-02-16

**Authors:** Jason Hill

**Affiliations:** 1 Science for Life Laboratory, Department of Medical Biochemistry and Microbiology, Uppsala University, Uppsala, Sweden

In the article "The genome sequence of the Mouse Moth, *Amphipyra tragopoginis* (Clerck 1759)", Boyes *et al.* report the genome assembly and gene annotation of a historically well-studied Lepidoptera. The genome sequencing and scaffolding methods are state of the art and the resulting assembly represents a solid resource for the Lepidoptera community.

 While it may be beyond the scope of the current program, a comparison of synteny relationships with other well studied Lepidoptera would be an interesting addition to this and future species. Given the high quality annotation this would be a further confirmation of the genome assembly quality.

Are sufficient details of methods and materials provided to allow replication by others?

Yes

Is the rationale for creating the dataset(s) clearly described?

Yes

Are the datasets clearly presented in a useable and accessible format?

Yes

Are the protocols appropriate and is the work technically sound?

Yes

Reviewer Expertise:

Genome assembly and annotation, Lepidoptera population genomics, structural biology, biochemistry.

I confirm that I have read this submission and believe that I have an appropriate level of expertise to confirm that it is of an acceptable scientific standard.
